# Strategies for Small Extracellular Vesicle-Based Cancer Immunotherapy

**DOI:** 10.34133/research.0421

**Published:** 2024-07-22

**Authors:** Yundi Chen, Shasha Tang, Fengfeng Cai, Yuan Wan

**Affiliations:** ^1^ Department of Breast Surgery, Tongji Hospital, School of Medicine, Tongji University, Shanghai, China.; ^2^ The Pq Laboratory of BiomeDx/Rx, Department of Biomedical Engineering, Binghamton University, Binghamton, NY, USA.

## Abstract

Extracellular vesicles (EVs) are lipid bilayer-enclosed vesicles released by cells. EVs encapsulate proteins and nucleic acids of their parental cell and efficiently deliver the cargo to recipient cells. These vesicles act as mediators of intercellular communication and thus play a crucial role in various physiological and pathological processes. Moreover, EVs hold promise for clinical use. They have been explored as drug delivery vehicles, therapeutic agents, and targets for disease diagnosis. In the landscape of cancer research, while strides have been made in EV-focused cancer physiopathology, liquid biopsy, and drug delivery, the exploration of EVs as immunotherapeutic agents may not have seen substantial progress to date. Despite promising findings reported in cell and animal studies, the clinical translation of EV-based cancer immunotherapeutics encounters challenges. Here, we review the existing strategies used in EV-based cancer immunotherapy, aiming to propel the development of this emerging yet crucial field.

## Introduction

Extracellular vesicles (EV) are lipid bilayer-enclosed submicrometer vesicles that are released from cells. Based on biogenesis and size, EVs are divided into 3 subtypes: exosomes, microvesicles, and apoptotic bodies, and EVs with a diameter smaller than 200 nm are additionally classified as small EVs (sEVs) [1]. EVs can selectively encapsulate proteins and nucleic acids of their parent cell [2]. Subsequently, EVs enter body fluids and deliver the cargo to adjacent and/or distant recipient cells via endocytosis, receptor–ligand interaction, or membrane fusion [3]. Upon completion of delivery, EV cargo can be released into cytosol or integrated onto plasma membranes [4]. The delivered cargo could reprogram the recipient cells and ultimately alter their phenotype [5]. These biofunctions are upheld by EV unique properties, including biocompatibility, stability, long circulation half-life, and deep tissue penetration, particularly for sEVs [6]. In brief, EVs play vital roles in pathophysiological processes, and relevant mechanistic studies are flourishing [7–9]. On the other hand, EVs hold promise for translational discoveries. As EVs inherit molecules from parental cells, isolating and detecting abnormal cell-derived EVs can aid in disease diagnosis [10,11]. As natural nanocarriers, EVs have been explored as drug delivery vehicles [12]. Recently, EVs carrying endogenous cargo are being tested as a new drug modality [13]. Reviews on specific topics, including EV biogenesis, physiopathology, isolation and detection techniques, diagnostics, drug delivery, and scalable manufacturing, are available elsewhere [14–17]. Here, we will refrain from repeating them further and concentrate on EV-based cancer immunotherapy.

Cancer immunotherapy’s success in inducing long-term remission and potential cures has substantially transformed the therapeutic landscape for cancers [18]. It is unsurprising that research on EVs has long been intertwined with cancer immunotherapy. Relevant investigations can be tracked back to 1998 [19]. EVs have been used as cancer vaccines to educate immune cells against tumors. Moreover, EVs derived from various donor cells, such as dendritic cells (DCs), tumor cells, and bacteria, have been tested for direct tumor treatment or as adjuvants in therapy. In summary, EV-based cancer immunotherapy holds several advantages over traditional approaches. Engineered EVs can precisely target cancer cells, reducing damage to healthy tissues. Through the delivery of immunostimulating molecules, they effectively modulate the immune system, bolstering the body’s natural defenses against cancer. Being naturally occurring, EVs are biocompatible and less likely to cause side effects compared to synthetic nanocarriers, especially when using autologous EVs. Their stability also enables them to circulate for extended periods, maximizing their therapeutic potential. Their small size facilitates penetration of physical barriers, augmenting their reach and efficacy. Furthermore, EV-based immunotherapies can even be combined with other treatments for synergistic effects and improved outcomes. While studies demonstrated success in cell and animal models, EV-based cancer immunotherapy still requires further development, especially when compared to the advancements seen in chimeric antigen receptor T (CAR-T) cell therapy and immune checkpoint blockade therapy. Current research on EV-based cancer immunotherapy remains fragmented and lacks systematic exploration. Nonetheless, the actuality presents significant opportunities for advancement in this field.

Hence, we conducted a review of current EV-based cancer immunotherapy strategies, aiming to advancing their development and clinical translation. Notably, our literature collection focused on EVs primarily used for cancer immunotherapy, such as cancer vaccine, adjuvants, and immune modulation. We excluded literature where EVs were used for non-immunotherapy purposes but incidentally observed their impact on the host immune system. Keywords including exosomes, EVs, cancer, immunotherapy, and others were used to search for relevant peer-reviewed articles across PubMed, Medline, and Google Scholar. To ensure the inclusion of recent findings, we primarily included studies published within the last 10 years. Unlike existing reviews that delve deeper into mechanisms, donor cell sources, or EVs as delivery vehicles for immunotherapeutic agents [20–24], this review focuses on the practical considerations of EV-based cancer immunotherapy for bioengineers and oncologists. We prioritized developed strategies, achievements, and remaining challenges. The summarized information can inspire bioengineers and oncologists in building upon existing knowledge and developing new technologies. Additionally, we concluded by discussing future directions and hurdles in this field.

## EVs Used in Cancer Immunotherapy

The diverse cell sources of EVs frequently used in cancer immunotherapy are summarized (Table 1). More information is available elsewhere [12]. Notably, a variety of cell strains and methods are being developed or used for production of EVs [25,26]. This trend is evident with the ever-increasing development of engineered cells and derived EVs [27,28]. Therefore, rather than presenting an exhaustive list of all cell sources, we generalize their characteristics. It is apparent that EVs originating from diverse sources exhibit distinct immune-related and tumor-related characteristics, thereby influencing aspect of cancer immunotherapy strategy, efficacy, cost, consistency, and regulatory considerations [29–33]. For example, the biocompatibility of EVs significantly influences their ability to elicit immune responses and the magnitude thereof [34]. In addition, EVs obtained from immortalized cell lines, as opposed to non-immortalized ones, may offer enhanced consistency and can be manufactured at a larger scale with more affordable costs [35]. In summary, considering these criteria and practical needs, selection or construction of donor cells is essential when developing EV-based cancer immunotherapy.

**Table 1.** Commonly used EVs in cancer immunotherapy

EV sources	Immune function	Targeting	Advantages	Disadvantages
Tumor cell-derived EVs (TEV) [172]	• Delivery of tumor-associated antigens to APCs • Induction of cytokine release and chronic inflammation • Involvement in the formation of the immunosuppressive tumor microenvironment	• Homing effect	• Rich of tumor antigens • Autologous • High yield	• Tumor-promoting molecules containment
Dendritic cell (DC)-derived EVs [173–175]	• Activation of T cells, B cells, and NK cells via direct MHC-I/MHC-II contact • Indirect stimulation of T cells through bystander DCs • Activation of NK cells and T_H_ cells via NKG2D-L and the IL-15/IL-15Rα complex • Cytokine release	• MHC binding • Receptor–ligand binding	• Antigen-presenting • Rich of cytokine • Autologous	• Immunogenicity • Low yield • High batch-to-batch variation • Substantial variance in treatment effectiveness
T cell-derived EVs [176–179]	• Cytotoxic effect (e.g., CD4^+^ T cell and CAR-T) • Immunomodulatory (e.g., CD8^+^ T cell) • B cell activation (e.g., CD4^+^ T cell)	• Immune recognition • Receptor–ligand binding	• Selective cytotoxicity • Autologous	• Low yield • Donor cell-dependent functionality • High batch-to-batch variation • Safety issue
Macrophage-derived EVs [180]	• M1-derived EVs carrying MHC and CD54, crucial for CD4^+^ or CD8^+^ T cell activation • M2-derived EVs promoting tumor invasion and metastasis	• Homing effect • MHC binding • EPR effect	• Antigen-presenting • Immune modulation • Autologous	• Low yield • High batch-to-batch variation
NK cell-derived EVs [181,182]	• Exertion of cytotoxic effects on tumor cells • Activation of other immune cells	• Receptor–ligand binding	• Potent cytotoxicity • Immune modulation • Autologous	• Low yield
Mesenchymal stem cell-derived EVs (MSC-EV) [183]	• Anti-inflammatory effect in tumor microenvironment • Immune cell recruitment	• Homing effect	• Immune modulation • High yield • Autologous	• High batch-to-batch variation
Apoptotic bodies (ApoBD) [184–186]	• Anti-inflammatory effect • APC stimulation	• “Eat me” signal • EPR effect	• Enhance macrophage uptake • Anti-inflammatory potential	• Complex components • Safety issue • Rapid immune clearance
*E. coli* outer membrane vesicles (OMVs) [105–107,187]	• High immunogenicity • APC stimulation • IFN-γ-based antitumor effect • Cytokine storm at high dose	• “Eat me” signal • Immune recognition	• Excellent engineering potential • High yield Immune stimulation	• Endotoxin • Immunogenicity • Safety issue
HEK293(T)-derived EVs [188,189]	N/A	• Targeting ligands	• Excellent engineering potential • High yield	• Immunogenicity • No immune function

APC, antigen-presenting cell; MHC, major histocompatibility complex; EPR, enhanced permeability and retention

To optimize cancer immunotherapy with EVs, current research prioritizes development of EVs with precise cancer targeting, enhanced cargo loading and delivery, boosting anticancer immunity, and combination therapies, as the relevant challenges have not been fully addressed or developed yet. Therefore, improving EV efficacy in cancer immunotherapy remains a significant long-term research challenge. Additionally, scalable production of EVs and regulatory considerations are crucial aspects that require attention. In our humble opinion, future EV development for clinical translation favors genetic modification of donor cells and derived EVs to embed built-in targeting moieties and endogenous therapeutic cargo, avoiding the need for post-production modification and loading of exogeneous agents. Cancer targeting can be readily achieved by chemical, physical, or genetical modification. However, chemical and physical approaches involve multiple steps, such as conjugation, compulsory capping reactions, and repeated purification. Relevant solvents may impair the stability and antigen-binding capabilities of the conjugated targeting moieties. The lengthy procedure often results in payload leakage, detachment of targeting moieties, and product loss. Consequently, these chemically or physically modified EVs are less appealing to industrial manufacturers due to the low yield, high production cost, and potential batch-to-batch variation. In contrast, genetical modification is preferred. By modifying the donor cells, manufacturers can continuously harvest EVs with built-in targeting capabilities and immunotherapeutic cargo directly from cell culture supernatant. This “one-and-done” approach simplifies production and improves consistency. On the other hand, post-production modification and exogeneous agent loading encounter regulatory hurdles. Typical concerns, such as endogenous cargo depletion, the purity of the modified EVs in the total exogeneous agent loading capacity and efficiency, and other uncertainties, may give regulators pause. On the contrary, intact EVs directly collected from supernatant without additional surface modification or exogenous cargo loading can be regarded as biological products and may be eligible for potential clinical translation. Regarding donor cell sources, the multifaceted influence of tumor cell-derived EVs (TEVs) on tumors complicates their use in the clinic. Engineered stem cells, immune cells, or readily available “normal” cells could be a safer option; however, demonstrating their efficacy and long-term safety remains paramount.

## Strategies for EV-Based Cancer Immunotherapy

EVs have been used to deliver and/or present tumor-associated antigens (TAAs), immune adjuvants, immunomodulators, and immune checkpoint inhibitors. Moreover, EVs are being combined with oncolytic virus (OV), CAR-T cells, and CRISPR/Cas9 system for cancer immunotherapy. In general, these strategies leverage EV’s biocompatibility, stability, tissue penetration, cargo capacity, and membrane fusion for therapeutic benefit.

### EV-based cancer vaccines

Cancer vaccines activate the immune system to recognize and attack tumors by stimulating it with specific antigens. Cancer vaccine immunotherapy offers distinct advantages due to its highly specific tumor cytotoxicity, robust memory T cell response, high therapeutic effects, and minimal systemic toxicity [36–38]. EV-based cancer vaccines represent the most extensively studied aspect of EV cancer immunotherapy. Currently, EVs are used to induce immune responses by presenting TAAs; alternatively, EVs can deliver adjuvants that can nonspecifically elicit immune responses [39].

#### TAA presenting

TAAs, present on tumor cells and to a lesser extent on some normal cells, are commonly used in cancer vaccines [40]. Primarily, TEVs as shuttles deliver TAAs directly to antigen-presenting cells (APCs), such as DCs. The delivery efficiently primes the immune system to recognize and subsequently eradicate TAA-expressing tumor cells via cytotoxic T lymphocytes (CTLs) while sparing normal cells. Moreover, TEVs may sculpt the tumor microenvironment, enhance the function of other immune cells including CD4^+^ T cells, natural killer (NK) cells, macrophages, and B cells, and induce the apoptosis of tumor cells. Although the precise EV-induced tumor-killing mechanisms remain under investigation, this knowledge gap does not necessarily impede their potential applications. At present, TAA presentation strategies predominantly encompass (a) using TAA-expressing TEVs to educate the immune system directly, (b) expressing TAAs on or loading them into EVs secreted by other donor cells to stimulate the immune system, and (c) using vector expression systems to generate TAA-bearing EVs in situ, thereby stimulating the immune system (Figure A to C). Meanwhile, to induce sufficient immune responses, neoantigens, fusion proteins, and adjuvants can be incorporated into EVs. The versatility of EVs is further amplified by the ability to graft targeting ligands onto their surfaces. This strategy allows for the specific delivery of TAAs to APCs via receptor-mediated interactions, significantly improving targeting efficiency.

**Figure.**
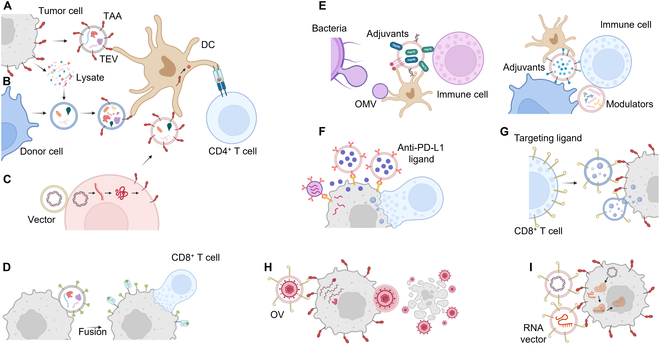
Existing strategies for sEV-based cancer immunotherapy. (A) TEVs as cancer vaccines directly present/deliver TAAs to APCs. (B) Tumor cell lysate-loaded EVs as cancer vaccines present/deliver TAAs to APCs. (C) TAA-expressing EVs produced from transfected donor cells as cancer vaccines present/deliver TAAs to APCs. (D) EVs as cancer vaccines confer TAAs or neoantigens onto tumor cell surfaces, triggering host immunity specifically against TAA- or neoantigen-expressing cancer cells. (E) EVs and OMVs as nanocarriers deliver adjuvants and/or immunomodulators, boosting immune responses. (F) Anti-PD-L1 ligand-expressing EVs or OMVs as immunotherapeutic agents block PD-L1, achieving immune checkpoint blockade. (G) CAR-T EVs as immunotherapeutic agents deliver GrB and other cytotoxins to tumor cells, inducing tumor apoptosis. (H) EVs as nanocarriers facilitate cytosolic delivery of OVs, enhancing tumor cell lysis. (I) EVs as nanocarriers deliver plasmid-encoding CRISPR-Cas9 and guide RNA (gRNA), enabling endogenous generation of CRISPR-Cas9 complex for gene editing in tumor cells.

During the formation of TEVs, a broad spectrum of TAAs can be loaded, thereby enabling TEVs to provoke immune responses (Figure A) [41]. TEVs can be harvested from cell culture supernatant or patient’s body fluids, such as plasma, hydrothorax, and ascites. Early studies demonstrated that TEVs can induce DC-mediated CTL activation [42]. Moreover, TEVs can elicit stronger immune responses compared to irradiated tumor cells, apoptotic bodies, or tumor lysates [42,43]. Although robust antitumor immune responses can be induced in animal models [44–50], the use of TEVs in clinical trials, if feasible, may offer limited benefits. TEVs carry immunosuppressive molecules, such as transforming growth factor-β (TGF-β) and PD-L1, which can dampen immune activation and promote immune tolerance. Additionally, TEVs may express self-antigens that are recognized as “self” by immune systems, leading to immune evasion. To boost immune responses induced by TEVs, an effective strategy involves co-presenting TAAs and neoantigens on EV surface. EVs from engineered B16 melanoma cells, expressing both TAAs and antigens of *Mycobacterium tuberculosis*, reduced tumor volume in mice by 80% compared to controls [51]. While TEVs can potentially function as cancer vaccines, they paradoxically foster tumor growth and metastasis, raising safety concerns about their use [52]. Indeed, due to these risks, the use of TEVs in clinical trials is uncommon.

Alternatively, for safety considerations, EVs from immune cells or stem cells have been investigated for loading and presenting TAAs (Figure B) [53,54]. γδ-T EVs preloaded with Epstein–Barr virus transformed lymphoblastoid cell lysates (as TAAs) enhanced tumor-specific T cell responses in vivo and increased survival rate to 80% compared to 5% for free lysates at 100 days after inoculation [55]. In another 2 studies, ovalbumin (OVA)-expressing EVs derived from T cells express major histocompatibility complex class I (MHC-I), CD80, CD40L, and interleukin-2 (IL-2); they can stimulate OVA-specific CTL responses by counteracting CD4^+^25^+^Foxp3^+^ T regulatory (T_reg_) suppression [56,57]. Mature DC EVs possess peptide–MHC-I and peptide–MHC-II complexes [58], and they are resistant to immunosuppressive tumor microenvironment [59]. Therefore, mature DC EVs are frequently used as cancer vaccines. In contrast, immature DC EVs lack the necessary antigen-presenting capacity and immunostimulatory properties. It was reported that HER2-expressing DC EVs can stimulate HER2-specific CTL responses. However, these HER2-expressing EVs only led to partial protective immunity in double-transgenic HLA-A2/HER2 mice with self-immune tolerance to HER2 [60]. To further enhance immune responses, human HER2 and rat neu fusion protein-expressing DC EVs were developed [61]. HER2/neu-expressing EVs stimulate enhanced CD4^+^ T cell responses, leading to increased induction of HER2-specific antibody compared to that triggered by the HER2-expressing EVs. In addition, HER2/neu-expressing EVs stimulate stronger HER2-specific CTL responses, eradicating 90% of HER2-overexpressing 4T1 tumor cells, while HER2-expressing EV-induced CTL responses only eliminate 53% of targets. OVA-loaded mature DC EVs transformed immunosuppressive macrophages into immunostimulatory ones, enhanced antigen presentation, and activated both innate and adaptive immune responses. Mice treated with OVA-loaded mature DC EVs exhibited a higher survival rate (~40% at day 30) compared to those receiving OVA-loaded immature DC EVs (0% at day 20) [62].

Stem cell-derived EVs have also been tested in cancer immunotherapy. Their cargo, such as miRNAs and cytokines, can modulate immune responses. Additionally, stem cells can uptake and process TAAs. The derived EVs can present TAAs to APCs [63]. Embryonic stem cell-derived EVs expressing embryonic antigens (EAs) and loaded with granulocyte-macrophage colony-stimulating factor (GM-CSF) significantly suppressed Lewis lung carcinoma growth. Tissue analysis revealed robust CTL responses and favorable immune profiles [T helper 1 (T_H_1) cytokines and T_reg_ down-regulation]. This translated to improved survival (60% at day 44 versus 0% control at day 24 ) and decreased tumor growth (~3-fold change at day 27) in the EV vaccine group [64]. The succeeding study further demonstrated that GM-CSF-expressing EVs inhibit tumor growth and metastasis [65]. These EVs reduced the lung tumor burden from 1.86% in nonvaccinated mice challenged with LLC to 0.0035% in corresponding vaccinated mice. Induced pluripotent stem cells (iPSCs) expressing a broad spectrum of TAAs have been constructed [66]. The derived EVs mixed with DCs induced antitumor immune responses both in vitro and in vivo, leading to effective elimination of a variety of tumor cells, including melanoma, lung cancer, breast cancer, and colorectal cancer. Notably, while stem cell-derived EVs demonstrated their safety and effectiveness, a recent study reported that stem cell-derived EVs might be less effective than TEVs in terms of the intensity and efficiency of antitumor immune responses [67]. Stem cell EVs may have both tumor-suppressive and tumor-promoting properties, depending on the type of stem cells and the cargo [63]. It may explain the failure of stem cell-derived EVs to elicit the desired anticancer immune responses.

Bypassing the laborious preparation of TEVs and TAA-loaded/TAA-expressing EVs derived from other donor cells, recent studies simplified the process by injecting vectors encoding TAAs, inducing the endogenous production of TAA-expressing EVs and subsequent immune responses (Figure C). Injecting CD63-OVA plasmids included the production of OVA-expressing EVs, which activated CTLs in immunized mice [68]. This response was not observed with the OVA plasmid alone. Moreover, endogenously generated EVs expressing mutated HIV-1 Nef/HPV16-E7 fusion proteins exhibited high antitumor efficiency, resulting in complete eradication of tumors expressing HPV antigens. The tumor-free mice showed long-term immune memory, with no tumor recurrence even at 130 days after administration [69]. Given that an efficient immune response often requires a substantial quantity of TAAs [70], researchers explored endoplasmic reticulum stress in donor cells to increase the payload of TAAs. Meanwhile, the derived EVs exhibited reduced immunosuppression and increased levels of immune adjuvants. The combined effects led to robust DC activation both in vitro and in vivo [71]. Using vectors for in situ EV vaccine generation is an intriguing concept with the potential for targeted and personalized cancer immunotherapy. However, host’s immune system may recognize and mount immune responses against the vector itself, particularly after multiple administrations, which can hinder its ability to deliver the instructions for creating endogenous EV vaccines.

As described above, EVs have proven effective in presenting TAAs to immune cells, triggering tumor elimination. In the latest study, researchers explored the direct conferment of HER2 on EV membranes onto HER2-null tumor cell membranes (Figure D) [72]. In light of this, the potential exists for neoantigens to be incorporated into tumor cell membranes via EV/cell membrane fusion, facilitating recognition and specific clearance by the immune system. To improve the efficiency of conferring TAAs onto recipient cell membranes and prevent lysosomal degradation of TAA-expressing EVs [73], viral fusogen-functionalized EVs have been developed [74]. In an acidic environment, the efficiency of EV–cell membrane fusion efficiency achieved ~65% within 30 min, facilitating TAA transfer [75]. Given this premise, TAA-loaded EVs expressing fusogens and targeting ligands could efficiently deliver TAAs to APCs.

#### Delivery of adjuvants

Non-antigenic immune adjuvants play a crucial role in cancer immunotherapy. They boost immunogenicity and antigen presentation, enhancing responses to weakly immunogenic tumors. Previous studies have demonstrated that adjuvant-loaded EVs exhibit superior efficacy compared to immunization with free adjuvants. Building on these findings and EV properties, EVs have been explored as nanocarriers to deliver adjuvants. In terms of loading, adjuvants can be loaded into EVs through methods, such as electroporation and sonication, although the loading efficiency still requires significant improvement. Alternatively, EVs derived from immune cells can directly function as adjuvants, modulating immune cell proliferation and differentiation [76,77]. For precise targeting, researchers can either leverage inherent homing effects of EVs or engineer their surfaces with targeting ligands, done physically, chemically, or genetically [78–81]. Moreover, EV’s ability to deliver adjuvants directly into the cytosol via membrane fusion holds promise for enhanced immune stimulation [82]. To achieve desired immune responses, strategies have progressed from solely delivering adjuvants to co-delivering a mixture of TAAs and adjuvants. Overall, previous studies demonstrated that EVs efficiently deliver diverse adjuvants, including oligonucleotides, lipids, and cytokines, to APCs (Figure E). These adjuvants can be either attached to the outer membranes of EVs or encapsulated within the lipid bilayer, depending on whether they need to bind to membrane or cytoplasmic proteins of recipient cells. The existing reviews have already covered the relevant content of using EVs as adjuvant nanocarriers [22]. Here, we will focus on introducing common adjuvants used in cancer immunotherapy, their associated effects, and potential concerns.

CpG DNA is a popular nucleic acid adjuvant. CpG refers to a DNA sequence containing cytosine followed by guanine [83]. Approximately 80% of cytosines at CpG sites in mammalian genomes are methylated [84]. Its unmethylated form can activate Toll-like receptor 9 (TLR9), triggering the activation of immune cells and cytokine secretion, e.g., interferons and IL-2, thereby promoting the maturation and activation of DCs and enhancing antitumor immune responses [85]. Moreover, DNA adjuvants can facilitate the uptake and processing of antigens by DCs, leading to increased presentation of antigenic peptides to T cells, promoting the activation of adaptive immune responses. CpG oligonucleotide ODN 1668-loaded B16BL6 cell-derived EVs resulted in increased cytokine release from DC2.4 cells, enhanced antigen presentation, and stronger immune responses in comparison to free CpG or EVs alone [86]. It is noteworthy that TLR9 is typically located within the endosomal compartments of DCs, where it recognizes unmethylated CpG motifs, inducing immune responses. These oligonucleotides could be degraded by nucleases, thereby impairing effectiveness.

Despite diverse mechanisms, lipid adjuvants effectively stimulate antigen responses. Bovine serum-derived EVs were first grafted with a conjugate, 1,2-distearoyl-sn-glycero-3-phosphoethanolamine-PEG-α-d-mannose, and then loaded with monophosphoryl lipid A (MPLA), a TLR4 agonist [87]. These MPLA-loaded EVs demonstrated enhanced targeting toward DCs, ultimately leading to increased cytokine release. Moreover, these EVs accumulated in high numbers within lymph nodes [88]. Another example of a lipid adjuvant is ISCOMATRIX, which uses purified viral lipids and boasts a safety record in human trials [89]. The co-delivery of mutant HIV-1 Nef-expressing human embryonic kidney (HEK) 293T EVs with ISCOMATRIX resulted in a notable increase in the pool of CTLs and significantly enhanced antigen cross-presentation [90]. It is important to note that lipid A, the biologically active component of lipopolysaccharide, also known as endotoxin, is responsible for eliciting toxic responses in the host immune system. Accordingly, development of lipid A-incorporated EV vaccines necessitates judicious optimization of dose, formulation, route of administration, and timing of administration.

Cytokine adjuvants stand out for their versatility in immune modulation, biocompatibility, and ability to fine-tune immune responses with manageable side effects. Their use in cancer immunotherapy dates back to the 1970s with successful applications like IL-2 for advanced melanoma [91]. While standalone IL therapy often falls short, IL-loaded EVs hold promise as antitumor agents. IL-2-expressing TEVs promoted T cell proliferation and enhanced the efficiency of antigen-specific responses in CTLs in vitro [92]. IL-12-loaded TEVs effectively inhibited tumor development, independent of anti-TGF-β1 short hairpin RNA (shRNA) treatment [93]. Beyond IL-2 and IL-12, GM-CSF is another well-studied cytokine adjuvant with potential for incorporation into EV-based cancer immunotherapy. GM-CSF influences various immune cells, particularly macrophages, DCs, and eosinophilic granulocytes, which translates to enhanced antitumor immune responses. RenCa cell-derived EVs loaded with GM-CSF and IL-12 adjuvants activated CTLs via the FasL/Fas signaling pathway. The in vivo tumor model showed an increased CD8^+^/CD4^+^ T cell ratio, indicating enhanced antitumor cytotoxicity [94]. A phase 1 trial investigated autologous ascite-derived EVs loaded with GM-CSF for colorectal cancer. This approach was safe and induced significantly higher tumor-specific CTL responses (76.9% versus 20%) compared to EVs alone [95]. In brief, loading cytokines into EVs offers several compelling advantages. This approach can mitigate systemic toxicity associated with cytokine therapy, enable targeted delivery to tumors, and promote localized immune activation within the tumor microenvironment. The co-delivery of TAAs and cytokines via EVs could further boost immune responses by increasing antigen presentation and T cell activation. Nevertheless, many cytokines have short half-lives in vivo, requiring frequent administration. This shortcoming can be logistically challenging.

Heat shock proteins (HSPs), particularly Hsp70 and Hsp90, act as adjuvants by stimulating DCs and up-regulating the expression of surface proteins, such as CD83, CD80, and CD86. Consequently, T cell and NK cell proliferation can be promoted, leading to enhanced tumor-specific and general cytotoxic responses [96,97]. Moreover, HSP can bind tumor-derived peptides, forming multivalent complexes that enhance immune recognition and potentially prevent tumor immune escape [98]. Notably, research also suggests a conflicting role for HSP in promoting immune tolerance and tumor progression [99,100]. Nevertheless, a recent study reported that pancreatic cancer-derived EVs carrying Hsp70 can activate NK cells and induce tumor apoptosis through released granzyme B (GrB) and perforin [101,102]. DC EVs loaded with chaperone-rich cell lysates (CRCLs) rich in Hsp70/90 significantly boosted CD4^+^ and CD8^+^ T cell activity in vitro compared to unloaded EVs. In vivo, these CRCL-loaded EVs enhanced T cell infiltration in glioma tumors, leading to suppressed tumor growth and extended survival (60% at day 60 versus 0% at day 35) [103]. Moreover, heat shock therapy, by elevating HSP levels in tumors, could synergize with HSP adjuvant therapy to further amplify the antitumor immune response. Hsp70-expressing EVs loaded with tellurium nanoparticles exhibited promise as a dual-action therapy: (a) stimulating immune response via Hsp70 and (b) enabling light-mediated tumor destruction. These EVs induced significant immunogenic death of tumor cells, improved DC maturation*,* and led to tumor eradication in 60% of mice [104]. While HSPs offer several advantages as cancer immunotherapy adjuvants, the source of HSPs may lead to variable immunogenicity. The efficacy of HSP-based EV vaccines in cancer immunotherapy also remains to be fully elucidated. In addition, HSPs may trigger autoimmune responses or nonspecific immune activation. In general, the use of HSPs as adjuvants is relatively uncommon compared to other adjuvants.

Gram-negative bacterial outer membrane vesicles (OMVs), enriched with outer membrane proteins, are being explored as adjuvants or vaccines due to their immunogenicity, a favorable safety profile, and stability. OMVs can specifically interact with DCs via polysaccharide-TLR2, stimulating regulatory T cells and cytokine production [105]. Moreover, OMVs can activate APCs by delivering bacterial peptides through internalization by epithelial cells [106]. Studies have demonstrated that OMVs can effectively indue long-term antitumor immunity with minimal side effects [107]. A recent study explored 1-methyl-tryptophan (1-MT), an inhibitor of indoleamine 2,3-dioxygenase (IDO), loaded into maleimide group (Mal)-grafted OMVs (1-MT@OMV-Mal) for cancer therapy. These 1-MT@OMV-Mal captured tumor antigens after photothermal therapy and enhanced DC uptake. In situ injection of 1-MT@OMV-Mal effectively countered IDO-mediated T cell suppression and significantly inhibited both primary and distant tumors, demonstrating a 2-fold superior effect compared to OMVs alone [108]. In another study, fusion protein SIRPα–Fc expressing OMVs were loaded with GM-CSF for cancer therapy. These OMVs promoted T cell-mediated immunity in the MC38 tumor model (tumor-associated macrophage-hot and T cell-cold) and antibody-mediated immunity in the B16-F10 tumor model (T cell-hot and tumor-associated macrophage-cold) [109]. Recently, co-administration of melanoma EVs with engineered OMVs achieved significant tumor regression (~65%) and prolonged survival (18 days versus 24 days) via a T_H_1-type immune response [110]. The main concern is the immune tolerance of OMVs. The prolonged administration of OMVs may induce immune tolerance or immune clearance, which could limit their adoption in clinical practice. To mitigate this concern, responsive nanocloak decorated OMVs were developed recently [111]. Biocompatible manganese oxide (MnO_2_) nanoparticles were used to cloak OMVs from *Bacteroides fragilis*. This nanocloak strategy programmed the OMVs’ immunomodulating properties, resulting in the creation of nanocloaked OMVs with a pH-sensitive response. Upon internalization by DCs, the acidic lysosomal environment dissolved the nanocloak. This released Mn^2+^ ions and potently activated DCs, leading to significant inhibition of bacterial growth within tumors. Researchers further investigated these OMVs’ potential for both preventive and combination therapy with an anti-PD-L1 antibody against breast cancer. Pre-immunization with OMVs effectively protected healthy mice from tumor formation by preventing *B. fragilis* infections. In models of *B. fragilis*-infected breast tumors, these OMVs efficiently suppressed tumor-associated bacteria in both the primary tumor and lung metastases. This effect synergized with the antitumor efficacy of anti-PD-L1 antibody treatment.

Overall, EV delivery of adjuvants for cancer immunotherapy holds promise but faces challenges. Careful adjuvant selection is crucial, prioritizing activation of CD4^+^/D8^+^ T cells to drive a T_H_1-type immune response. Optimizing encapsulation methods, adjuvant type, and stability of loaded adjuvants is compulsory, as these factors significantly impact the final immune responses. Improving loading capacity and efficiency is also essential for its successful clinical application. In addition, safety concerns remain, as adjuvants can induce inflammatory reactions, such as fever, ulcer, or even life-threatening cytokine storm. Moreover, investigating adjuvant-loaded EVs under clinically relevant conditions is also crucial to translating this approach.

### EVs as immunomodulator nanocarriers

In addition to adjuvants, EVs can also deliver immunomodulators. Small RNAs (smRNA), such as miRNA and small interfering RNA (siRNA), hold promise for cancer immunotherapy due to their targeted gene silencing via RNA interference. But its effectiveness can be limited by tumor heterogeneity, immune suppression, and potential off-target effects. EVs as nanocarriers can improve cytosolic delivery of smRNAs and protect them from degradation because of their liquid bilayer. Currently, 3 approaches have been developed for delivering smRNAs via EVs. (a) Exogeneous smRNAs can be loaded into EVs via mechanical methods. (b) Genetically modified donor cells can be engineered to express endogenous smRNAs, which are then sorted into EVs. (c) EVs with cancer immunotherapeutic potential can be directly harvested from stem cell culture supernatant. While optimizing loading efficiency remains an area of ongoing research, studies already demonstrated the effectiveness of smRNA-loaded EVs in cancer immunotherapy (Figure E). A study explored miR-130-loaded EVs derived from 4T1 cells to reprogram M2 macrophages to the antitumor M1 phenotype. These EVs up-regulated MA markers [CD86, Irf5, Nos2, tumor necrosis factor-α (TNF-α), and IL-1β] and down-regulated M2 markers (CD206, Ym1, Arg, TGF-β, and IL-10). Consequently, the reprogrammed macrophages inhibited the migration and invasion of 4T1 cells [112]. Another approach involves miR-155-loaded EVs derived from CT26 cells to enhance DC maturation and antibody presentation. miR-155 delivery significantly up-regulated MHC-II, CD86, CD40, and CD83 on DC membranes, indicating successful DC maturation. In addition, miR-155 increased the expression levels of IL12p70 and interferon-γ (IFN-γ), further promoting antitumor immunity [113]. A recent study explored a new approach using M1 macrophage-derived EVs loaded with anti-PD-L1 siRNA. To enhance delivery, these EVs were decorated with vesicular stomatitis virus glycoprotein, a pH-responsive viral fusogen. This approach effectively silenced PD-L1 expression, leading to increased CTL population and repolarization of M2 macrophages to the M1 phenotype, demonstrating the potential to overcome immune checkpoint suppression [114]. siRNA-loaded EVs can also modulate immune responses. L929 EVs carrying anti-TGF-β1 siRNA inhibited tumor development and induced apoptosis [115]. In another study, EVs containing anti-VEGFR (vascular endothelial growth factor receptor) siRNA showed higher antitumor effect and lower toxicity compared to free drug, apatinib, for lung metastatic osteosarcoma (25% survival at day 80 versus 0% at day 58) [116]. In addition to the exogeneous loading of smRNAs into EVs, engineered donor cells can directly secrete EVs containing endogenous smRNAs. In brief, donor cells undergo genetic modification, enabling specific smRNAs to be overexpressed and encapsulated into EVs through intracellular cargo sorting. For example, human miR-371b-5p sequence was inserted into the LAMP2A gene, enabling HEK293T cells to continuously generate EVs carrying endogenous miR-371b-5p [117]. In animal models of osteosarcoma, these engineered EVs inhibited tumor growth and extended overall survival. Furthermore, EVs derived from stem cells naturally containing certain smRNAs possess cancer immunotherapeutic effects. A study found that mesenchymal stem cell-derived EVs containing miR-133b can suppress glioma progression via Wnt/β-catenin signaling pathway [118]. Mesenchymal stem cell-derived EVs containing miR-3940-5p inhibits colorectal cancer metastasis by targeting integrin α6 [119]. Bone mesenchymal stem cell-derived EVs containing miR-512-5p can inhibit glioblastoma progression by targeting JAG1 [120]. miR-199a-expressing EVs from adipose tissue-derived mesenchymal stem cells [121], miR-30b-5p-expressing EVs from bone mesenchymal stem cells [122], miR-320a-expressing EVs from human umbilical cord mesenchymal stem cells [123], and many other examples indicate that these EVs from stem cells can be used for cancer immunotherapy. Despite the promise of stem cell-derived EVs in cancer immunotherapy, their heterogeneity and substantial cargo variations raise concerns about therapeutic efficacy and reproducibility. The same concern also exists with genetically modified donor cell-derived EVs that carry certain smRNAs.

Certain chemotherapeutic drugs can also modulate the immune system, but the precise mechanisms are not fully understood. Loading these drugs into EVs not only reduces side effects and achieves targeted delivery [124] but also unlocks their potential for enhanced immune modulation. Methotrexate (MTX) [125], a chemotherapeutic drug with immunomodulatory properties, was loaded into EVs derived from apoptotic tumor cells for cancer treatment. Patients with extrahepatic cholangiocarcinoma showed that infusion of these MTX-loaded EVs into the bile duct lumen triggered 2 waves of neutrophil mobilization and activation, including an antitumor phenotype in the neutrophils. This resulted in alleviated biliary obstruction in 25% of the patients [126]. In another study, autologous lung tumor-derived EVs loaded with MTX (ATMPs-MTX) exhibited tumor-specific localization in animal models. These EVs exerted cytotoxic effects on both tumor cells and tumor-associated macrophages while sparing T cells. Intrapleural infusion of ATMPs-MTX into patients demonstrated a favorable safety profile, with good tolerability and no severe (grade 3 or higher) adverse effects [127]. Curcumin, a natural antioxidant with anti-inflammatory and antitumor properties, is also being tested as immunomodulators for cancer therapy. A recent study investigated curcumin-loaded milk EVs against MCF-7 and MDA-MB-231 breast cancer cells, along with MCF-10A nontumorigenic cells. The findings indicated that curcumin specifically inhibited cancer cell proliferation by approximately 3-fold while showing minimal effect on MCF-10A cells, highlighting their potential for safe and effective cancer treatment [128]. In general, the immunomodulatory effect of chemotherapy falls short of its direct cytotoxic action. This effect also depends on diverse factors, such as cancer type, tumor microenvironment, and timing of administration. Despite these shortcomings, the immunomodulatory effect has some merit. Nonetheless, understanding the mechanisms underlying this effect can allow us to optimize treatment strategies for maximizing therapeutic benefit. Similarly, the success of EVs as nanocarriers for delivering immunomodulators hinges on improvements in loading capacity and efficiency in clinical applications.

### EVs as immune checkpoint inhibitors

Immune checkpoints are regulatory mechanisms that prevent the immune system from attacking healthy tissues. Tumors exploit these checkpoints to evade immune destruction. Humanized monoclonal antibodies (mAbs) effectively inhibit checkpoint proteins from interacting with their ligands, thereby enabling tumor-infiltrating CTLs to recognize and eliminate tumor cells. Despite their effectiveness, humanized mAbs targeting PD-1/PD-L1 face several limitations. Deglycosylation or other engineering processes can lead to mAb aggregation, triggering production of anti-drug antibodies (ADAs) that neutralize therapeutic effects and, in rare cases, cause adverse reactions [129]. Moreover, high-dose regimens needed for efficacy can induce moderate to severe on-target, off-tumor toxicities, potentially discouraging patients [130]. Engineered EVs expressing immune checkpoint proteins offer a feasible strategy to counteract this immune evasion and enhance immunotherapy. One study explored PD-1-expressing OMVs for cancer therapy (Figure F). PD-1-OMVs demonstrated superior antitumor effects in vivo compared to controls, including free αPD-L1, free OMVs, and mixture of OMVs with αPD-L1 [131]. To further enhance efficacy, researchers developed LyP1 peptide-modified OMVs (LOMVs) loaded with PD-1 plasmid for self-targeting and PD-L1 blockade in tumors. At 30 days after administration, the survival rate was still 60%, significantly higher than control (0% at day 14) [132]. In another study, EVs expressing high-affinity variant human PD-1 (havPD-1) exhibited robust antitumor activity (Figure F) [133]. Even without the supply of CTLs, these havPD-1-expressing EVs derived from engineered MDA-MB-231 cells exhibited biofunctions. They were able to inhibit MDA-MB-231 cell proliferation, migration, and invasion while also inducing apoptosis in cancer cells. The mechanisms of apoptosis and inhibitory effects may be related to the blocking of the PD-L1 signaling pathway, receptor-mediated endocytosis/degradation of PD-L1, and the cargo carried by the engineered EVs. Their combination with chemical compounds further improved treatment effectiveness. Notably, immunogold staining revealed an average of 19 havPD-1 molecules on EV membranes. Trypsin treatment reduced this number, but still leaving an average of 12 havPD-1 molecules per EV. The finding indicates that EVs can effectively protect membrane proteins against proteolytic cleavage. In addition, havPD-1 EV administration necessitated a dosing frequency of every 2 to 3 days, more frequent than the 1-month interval typical of mAb therapy. This may suggest that EVs more efficiently fulfill their therapeutic purpose in the body. Overall, the therapeutic EVs expand the portfolio of immunotherapeutic agents and deserve further development.

### EVs as substitutes for cell therapy

CAR-T EVs offer promise for cancer immunotherapy, potentially surpassing their parent cells. CAR-T EVs demonstrate deeper tissue penetration and higher cytotoxic activity (Figure G) [134]. CAR-T EVs are less susceptible to the immunosuppressive tumor microenvironment. Conversely, immunosuppressive factors like IL-10 have been shown to inhibit CAR-T cell functionality [135]. While exhibiting lower cytokine levels, CAR-T EVs possess over 20-fold more GrB. In a HER2-positive tumor model, both CAR-T cells and CAR-T EVs can trigger cytotoxic effect through caspase-3/7 pathway, although CAR-T EVs may require 3-fold more time to induce massive apoptosis [136]. Additionally, anti-HER2 and anti-EGFR (epidermal growth factor receptor) scFv coexpressing CAR-T EVs demonstrated antitumor efficiency in animal models. Importantly, these CAR-T EVs exhibited an improved safety profile compared to CAR-T cells, with minimal cytokine release [137]. While CTL-derived EVs possess cytotoxic properties, a key question regarding these EVs is their efficiency in delivering encapsulated GrB into the cytosol. Endocytosis is the primary uptake pathway of EVs, and over 70% of endocytosed EVs are relocalized into lysosomes [138,139]. It implies that lysosomal degradation is the primary fate for GrB wrapped in these CTL EVs, although cytosolic escape might occur [140]. The incorporation of fusogenic moieties onto CTL EVs might be a strategy to address this concern.

### EVs as boosters of OV therapy

OV therapy uses engineered viruses to infect and lyse tumor cells. These viruses can also express therapeutic genes and/or proteins to further enhance tumor cell killing. Compared to other therapies, OVs offer several advantages, including high killing efficiency, precise targeting, and minimal side effects [141]. A variety of OVs are entering into clinical trials, with some already demonstrating efficacy. For example, Imlygic, approved by US Food and Drug Administration (FDA) in 2015, treats unresectable melanoma lesions [142]. However, a major challenge is the emergence of autologous antibodies against the OVs after multiple administrations, which can significantly weaken treatment efficacy.

EVs act as both double-edged swords in viral infections. EVs can inhibit viral replication via antiviral cargo, such as IFN-α or APOBEC3G, or boost host antiviral immunity [143]. Conversely, EVs can facilitate viral spread by carrying viral nucleic acids and promoting their uptake by target cells through interactions with specific membrane proteins, such as CD81, CD9, and CD63 [144–146]. Additionally, EVs many contribute to a virus’s ability to evade the immune system [146]. Furthermore, EVs can transport other viral components, such as proteins, which can enhance viral infection and virulence. Studies have verified that EVs promote infection of various viruses, including OVs [147,148].

Despite the unclear mechanisms underlying the interaction between EVs and OVs, this does not deter their combined use in cancer immunotherapy. Encapsulating OVs within EVs was developed to enhance therapeutic efficacy. Researchers loaded recombinant adenovirus that can express extracellular domain of PD-1 (Ad5-P) into HEK293T EVs (Figure H). These Ad5-P-loaded EVs demonstrated improved infection efficiency in cell lines with low susceptibility to OVs. Animal studies further demonstrated prolonged survival for animals treated with these Ad5-P-loaded EVs [149]. These findings suggest that EVs may improve OV therapy by facilitating cellular entry without relying on receptors and providing protection from immune clearance. In another study, OVs and paclitaxel were co-loaded into A549 EVs. In lung cancer model, this approach significantly improved antitumor activity in vitro and in vivo compared to using these agents separately [150]. On the other hand, EV encapsulation facilitates OV administration, protecting from immune clearance of OVs. EVs allow for intravenous administration, reaching deeper tumors, and homing effect improves targeting specificity. This contrasts with traditional OV administration, which requires direct injection into the tumor site. For instance, the oncolytic adenovirus Ad5D24 LL/2 showed improved tumor targeting when encapsulated in EVs and delivered intravenously. This indicates that EV-based delivery preserves OV infectivity and effectiveness, paving the way for broader application of OV therapy [147]. Toward clinical translations, limited payload capacity of EVs and variable loading efficiency of OVs should be addressed. Moreover, EVs loaded with OVs could inadvertently spread the OVs to unintended sites in the body, potentially leading to off-target effects or systemic infection, given the limitations in achieving absolute tumor specificity with current OVs.

### EVs in combination with gene therapy

CRISPR/Cas9 gene editing holds promise for gene therapy, but efficient delivery methods are crucial. While viral vectors offer high transfection efficiency, they can trigger immune responses or insertional mutagenesis [151]. Nonviral vectors, including lipid nanoparticles and liposomes, may also induce unfavorable immune responses, leading to cargo leakage and rapid clearance. EVs’ biocompatibility, tissue penetration, and loading capacity make them attractive nanocarriers. EVs derived from anti-CD19-CAR-HEK293T cells were used to deliver CRISPR/Cas9 system-expressing plasmid targeting the MYC oncogene (Figure I) [152]. Because the membrane-anchored chimera can significantly enhance EV’s binding affinity and tumor specificity, these CAR-HEK293T EVs accumulated faster in tumors and efficiently delivered plasmids in vitro and in vivo. While 3 to 10 μg of plasmids were used for loading via electroporation, the loading capacity or efficiency was not reported or compared with the existing viral/nonviral vectors. Nevertheless, the CAR-HEK293T EVs eliminated malignant B cells, demonstrating its therapeutic potential. EVs as delivery nanocarriers offer a solution to delivery challenges associated with CRISPR-Cas9 or relevant vectors. The main concerns arise from the gene editing technique itself. The challenges include but not limited to inherent off-target effects, long-term safety of CRISPR-Cas9-based cancer therapy, and ethical and regulatory considerations. Moreover, cancer cells may develop resistance to CRISPR-Cas9-based therapies, such as mutations in the targeted gene or activation of compensatory pathways. Overcoming or circumventing resistance mechanisms is also essential. Furthermore, either the quantity of vector-loaded EVs that can be harvested or their purity within the total harvested EVs remains unclear. Therefore, we view its potential clinical application with cautious optimism.

## Conclusion and Perspectives

Despite the promising preclinical data, significant hurdles remain in translating EV-based immunotherapy to the clinic. Although numerous studies are underway, only a handful have reached clinical trials (Table 2), and the outcomes are frustrating. The bankruptcy of Codiak resulted in the termination of one clinical trial (NCT05375604). The lack of disclosed data from 3 completed trials further raises concerns about the overall effectiveness of EV-based immunotherapy. The outcomes of the 2 remaining ongoing trials, expected to report by the end of 2024, remain uncertain. On a technical level, several challenges have been observed. (a) EV cancer vaccines require abundant TAAs to elicit immune responses. However, evidence suggests that TAA-overexpressing EVs may increase the risks of immune suppression and tumor metastasis. (b) Even without considering the risk of EVs promoting tumor development, EV vaccines may not be capable of inducing sufficient immune responses. Clinical studies demonstrated that DC EVs can activate NK cells and enhance T cell tumor infiltration. However, they failed to elicit a detectable CD4^+^ or CD8^+^ T cell response [153–155]. This finding aligns with the observation that even FDA-approved DC-based vaccines have demonstrated limited clinical efficacy [156]. While the use of adjuvants may boost immune responses, they have not been used in clinical trials. (c) Even if EV vaccines efficiently stimulate adaptive immunity, tumor cells can still evade the immune clearance through various unexplored mechanisms, resulting in limited therapeutic efficacy. (d) Off-target effects, the unpredictable nature of immunomodulators, complex EV cargo, and interindividual variability in treatment response are common factors contributing to treatment failure. On a clinical practice level, additional challenges require our attention. (a) The heterogeneity of EVs necessitates identifying precise surface markers for efficient classification and selection of desired subtypes for immunotherapy. (b) The use of exogenous EVs entails potential safety concerns arising from immunogenicity. (c) Our understanding of the interaction between immune cells and EVs remains superficial. EVs play a multifaceted role in regulating functions of various immune cells, making the impact of EVs on the immune system often unpredictable. Hence, a full understanding of the immune system’s response to EVs is crucial. (d) The production of EVs in sufficient quantities for therapeutic applications remains a significant challenge. Scaling up production methods while maintaining consistency and quality needs further development. Notably, extensive research has been conducted into the production of clinical-grade EVs for treatment, ensuring adherence to good manufacturing practice standards [157,158]. This concerted effort underscores the commitment to developing safe and effective therapies that meet stringent regulatory criteria [159].

**Table 2.** Clinical trials of EV-based tumor immunotherapy updated in recent years

NCT number	Start date	Completion	Enrollment	Phase	Status	Diseases	Interventions	Strategy applied
NCT05559177	2022-9-1	2023-6-1	9	1	Recruiting	Recurrent or metastatic bladder cancer	Chimeric EVs	Cancer vaccine
NCT05375604	2022-6-28	2023-5-30	9	1	Terminated	Advanced hepatocellular carcinoma Gastric cancer metastatic to liver Colorectal cancer metastatic to liver	STAT6 antisense oligonucleotide-loaded EVs	Delivery of immunomodulators
NCT03230708	2017-5-1	2018-2-1	18	1/2	Unknown	Malignant ascites	MTX-loaded erythrocyte EVs	Delivery of immunomodulators
NCT02657460	2016-1	2019-12	90	2	Unknown	Malignant pleural effusion	MTX-loaded TEVs	Delivery of immunomodulators plus cancer vaccine
NCT01294072	2011-1	2024-11	35	NA	Recruiting	Colon cancer	Curcumin conjugated with plant EVs	Delivery of immunomodulators
NCT01159288	2010-5-19	2015-12-19	41	2	Completed	Non-small cell lung cancer	Tumor antigen-loaded DC EVs	Cancer vaccine

Note: Data from ClinicalTrials.gov

In light of the challenges we introduced, a paradigm shift toward innovative treatment strategies is also warranted for EV-based cancer immunotherapy. The ideal cancer immunotherapy would equip the body to recognize and eliminate tumor cells with precision, similar to CAR-T cell therapy or mAb therapy. Meanwhile, we need to trigger proportionate and controllable immune responses, which should be potent enough to induce effective tumor clearance but not too strong as to cause excessive side effects. In accordance with these requirements, current EV-based immunotherapy approaches are still working toward achieving this goal. The existing strategies predominantly focus on (a) using EVs to present TAAs to immune cells, along with the use of novel adjuvants to enhance immune stimulation, (b) engineering EVs for efficient loading and delivery of TAAs, adjuvants, immunotherapeutic agents, and immunomodulators to recipient cells, (c) developing engineered EVs as a direct form of immunotherapy, and (d) exploring combination of these approaches for potential synergistic effects. Through ongoing research, we anticipate that even more effective EV-based cancer immunotherapies can be developed. In contrast, the use of EVs to enable tumor cells to carry neoantigens, thereby activating the immune system for tumor cell elimination, appears to have received limited exploration to date. Given that the epigenetic modulating drugs enable tumor cells to express neoantigens, eliciting immune clearance, we should also be able to leverage EVs’ properties to induce tumor cells to specifically express neoantigens, thereby achieving targeted immune clearance. To achieve this goal, EVs could be loaded with mRNAs or plasmids, encoding neoantigens for targeted delivery to tumor cells. Alternatively, engineering EVs to directly fuse their membranes with tumor cells could introduce neoantigens on the tumor cell surface itself. Nevertheless, this is just a glimpse into the exciting possibilities of EV-based cancer immunotherapy.

In conclusion, overcoming these hurdles, we could accelerate the relevant clinical translation and unlock the full potential of EVs for improving patient outcomes. It is noteworthy that EVs extend their reach beyond cancer immunotherapy. Research is actively exploring the use of EVs derived from immune regulatory T cells and stem cells to suppress unwanted immune responses [160–171]. This versatility highlights the exiting possibilities of EVs in tuning the immune system, offering the potential to both stimulate and dampen immune activity depending on the therapeutic need.
